# A novel strategy to improve protein secretion via overexpression of the SppA signal peptide peptidase in *Bacillus licheniformis*

**DOI:** 10.1186/s12934-017-0688-7

**Published:** 2017-04-24

**Authors:** Dongbo Cai, Hao Wang, Penghui He, Chengjun Zhu, Qin Wang, Xuetuan Wei, Christopher T. Nomura, Shouwen Chen

**Affiliations:** 1 Hubei Collaborative Innovation Center for Green Transformation of Bio-Resources, College of Life Sciences, Hubei University, No. 368 Youyi Avenue, Wuchang District, Wuhan, 430062 Hubei People’s Republic of China; 2 College of Food Science and Technology, Huazhong Agricultural University, Wuhan, 430070 China; 3 Department of Chemistry, The State University of New York College of Environmental Science and Forestry (SUNY ESF), Syracuse, NY 13210 USA

**Keywords:** *Bacillus licheniformis*, Signal peptide peptidase, Protein secretion, *sppA*, *tepA*

## Abstract

**Background:**

Signal peptide peptidases play an important role in the removal of remnant signal peptides in the cell membrane, a critical step for extracellular protein production. Although these proteins are likely a central component for extracellular protein production, there has been a lack of research on whether protein secretion could be enhanced via overexpression of signal peptide peptidases.

**Results:**

In this study, both nattokinase and α-amylase were employed as prototypical secreted target proteins to evaluate the function of putative signal peptide peptidases (SppA and TepA) in *Bacillus licheniformis*. We observed dramatic decreases in the concentrations of both target proteins (45 and 49%, respectively) in a *sppA* deficient strain, while the extracellular protein yields of nattokinase and α-amylase were increased by 30 and 67% respectively in a strain overexpressing SppA. In addition, biomass, specific enzyme activities and the relative gene transcriptional levels were also enhanced due to the overexpression of *sppA*, while altering the expression levels of *tepA* had no effect on the concentrations of the secreted target proteins.

**Conclusions:**

Our results confirm that SppA, but not TepA, plays an important functional role for protein secretion in *B. licheniformis*. Our results indicate that the *sppA* overexpression strain, *B. licheniformis* BL10GS, could be used as a promising host strain for the industrial production of heterologous secreted proteins.

**Electronic supplementary material:**

The online version of this article (doi:10.1186/s12934-017-0688-7) contains supplementary material, which is available to authorized users.

## Background

Heterologous expression is an effective strategy to improve the production of secreted proteins. Many industrial enzymes (proteases, α-amylase, etc.) have been produced by heterologous expression in bacteria and fungi [e.g. *Escherichia coli*, *Bacillus* species, and *Saccharomyces*] [1–3]. Compared to other expression systems, *Bacillus* species are regarded as promising host strains with numerous advantages including: non-toxicity, convenience for gene modification and high yields of target proteins [4–6]. In particular, *Bacillus licheniformis* has been shown to be an effective host strain for protein production in previous studies [7, 8].

The genetic engineering of host strain to improve production is regarded as an efficient tactic due to its universality and efficiency [9, 10]. A number of examples of this effectiveness are available. For example, *B. licheniformis* BL10 was constructed as a highly efficient host strain for protein production by knocking out ten genes coding for extracellular proteases (Mpr, Vpr, AprX, Epr, Bpr, WprA, AprE, BprA), flagellin and amylase in *B. licheniformis* WX-02. Using this engineered *B. licheniformis* BL10, a previous study has demonstrated that nattokinase activity could be increased by 39% [7]. Also, the translocation elements (SecA, SecDF etc.), post-translocation chaperone PrsA and signal peptidase I have been engineered for production of recombinant protective antigen, α-amylase, subtilisin and Dsrs [9, 11–14].

In general, secreted proteins are extracellularly transported through three steps: protein synthesis, translocation, and release. Signal peptides play an important role in protein secretion, these peptide sequences are cleaved from the mature protein by signal peptidase I during the late stage of the secretion process. The cleaved remnant signal peptides are left in the cell membrane and could affect protein transportation [15–17]. Signal peptide peptidase (SPP) is a membrane-bound enzyme that uses a serine/lysine catalytic dyad mechanism to cleave the remnant signal peptides in the cellular membrane and aids in protein secretion [18, 19]. TepA and SppA have been previously identified as putative signal peptide peptidases in *B. subtilis*, and the concentrations of secreted target proteins decreased dramatically for a *sppA*-null strain, which suggested that SppA was involved in protein secretion in *B. subtilis* [18]. Despite the central role for SppA in protein secretion, prior to our study, there have been no studies focused on the improvement of protein secretion via overexpression of SPP.

In this study, both nattokinase and α-amylase were selected as prototype target proteins for secretion to evaluate the function of SppA and TepA in *B. licheniformis* BL10, derived from the native strain *B. licheniformis* WX-02 [7]. Our results identified and confirmed that SppA is the main SPP for protein secretion, and the concentrations of total extracellular proteins and target proteins increased significantly for a strain overexpressing *sppA*. As a result of these studies, the host strain *B. licheniformis* BL10GS was successfully constructed as a highly efficient platform for protein secretion.

## Methods

### Bacterial strains and plasmids

The bacterial strains and plasmids used in this study were listed in Table 1. *B. licheniformis* BL10 was employed as the original strain for gene modification, and *E. coli* DH5α was served as the host strain for plasmid construction. The expression vector pHY300PLK was used for constructing protein expression vectors, and T_2_(2)-Ori was applied for constructing the gene knockout vectors and integrating expression vectors in this study.

**Table 1:** The strains and plasmids used in this study

Strains	Relevant properties	Source of reference
*Escherichia coli*		
DH5α	*sup*E44 Δ*lac*U169 (f 80 *lacZ*ΔM15) *hsd* R17 *recA*1 *gyr*A96 *thi*1 *rel*A1	[7]
*Bacillus licheniformis*		
WX-02	Polyglutamate productive strain (CCTCC M208065)	CCTCC
BL10	WX-02(Δ*mpr*, Δ*vpr*, Δ*aprX*, Δ*epr*, Δ*bpr*, Δ*wprA*, Δ*aprE*, Δ*bprA*, Δ*hag*, Δ*amyL*)	[7]
BL10/pHY-amyL	BL10 harboring pHY-amyL	This study
BL10/pP43SacCNK	BL10 harboring pP43SacCNK(CCTCC M2014253)	CCTCC
BL10T	BL10(Δ*tepA*)	This study
BL10S	BL10(Δ*sppA*)	This study
BL10T/pHY-amyL	BL10T harboring pHY-amyL	This study
BL10S/pHY-amyL	BL10S harboring pHY-amyL	This study
BL10T/pP43SacCNK	BL10T harboring pP43SacCNK	This study
BL10S/pP43SacCNK	BL10S harboring pP43SacCNK	This study
BL10GT	Overexpression of *tepA* in BL10	This study
BL10GS	Overexpression of *sppA* in BL10	This study
BL10GT/pHY-amyL	BL10GT harboring pHY-amyL	This study
BL10GS/pHY-amyL	BL10GS harboring pHY-amyL	This study
BL10GT/pP43SacCNK	BL10GT pP43SacCNK	This study
BL10GS/pP43SacCNK	BL10GS pP43SacCNK	This study
*Plasmids*		This study
T_2_(2)-ori	*Bacillus* knockout vector; Kan^r^	[7]
T_2_-sppA	T2(ori)-sppA(A + B); to knock out *sppA*	This study
T_2_-tepA	T2(ori)-tepA(A + B); to knock out *tepA*	This study
T_2_-GsppA	T2(ori)-sppA(A + B+*sppA*); to over-express *sppA*	This study
T_2_-GtepA	T2(ori)-tepA(A + B+*tepA*); to over-express *tepA*	This study
pHY300PLK	*E. coli* and *B. s* shuttle vector; Amp^r^, Tet^r^	[7]
pP43SacCNK	PHY300PLK + Promotor-P43 (*B. subtilis* 168) + signal peptide of SacC (*B. subtilis* 168) + *aprN*(*B. subtilis* MBS 04-6) + Terminator of *amyL* (*B. licheniformis* WX-02)	[7]
pHY-amyL	PHY300PLK + Promotor-P43 (*B. subtilis* 168) + *amyL* (containing its own signal peptide and terminator, *B. licheniformis* WX-02)	This study

### Media and culture conditions

The LB medium was served as the basic medium for bacterial growth, and the corresponding titer of antibiotic (50 μg/mL ampicillin, 25 μg/mL tetracycline or 20 μg/mL kanamycin) was added into the medium if necessary. The ME medium for cell growth contains 20 g/L glucose, 10 g/L sodium citrate, 7 g/L NH_4_Cl, 0.5 g/L K_2_HPO_4_·3H_2_O, 0.5 g/L MgSO_4_·7H_2_O, 0.04 g/L FeCl_3_·6H_2_O, 0.104 g/L MnSO_4_·H_2_O, 0.15 g/L CaCl_2_·2H_2_O. The inoculum (3%) was added into 30 mL of ME media in a 250 mL flask, and was incubated at 180 rpm and 37 °C for 24 h. All the fermentation experiments were repeated at least three times.

The medium for α-amylase production was supplied as follow: 5 g/L corn starch, 5 g/L yeast extract, 10 g/L peptone, 12 g/L sodium citrate, 1 g/L K_2_HPO_4_·3H_2_O, 0.5 g/L MgSO_4_·7H_2_O, 0.15 g/L CaCl_2_·2H_2_O, pH 7.2; The medium for nattokinase production containing 20 g/L glucose, 10 g/L soy peptone, 10 g/L peptone, 10 g/L corn starch, 15 g/L yeast extract, 10 g/L sodium chloride, 3 g/L K_2_HPO_4_·3H_2_O, 6 g/L (NH_4_)_2_SO_4_, pH 7.2. The inoculum (3%) was added into 30 mL of fermentation media in a 250 mL flask, and was incubated at 180 rpm and 37 °C for 48 h. All the fermentation experiments were repeated at least three times.

### Gene knockout in *B. licheniformis*

**Fig. 1:**
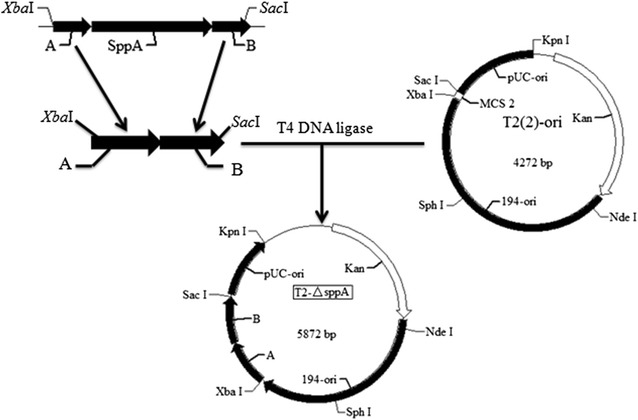
The construction procedure of gene knockout vector.* A* and* B* represented the* upstream* and* downstream* homologous arms of *sppA*, respectively

The genes *sppA* and *tepA* were deleted individually in *B. licheniformis* BL10 according to our previously reported method [20, 21], and the construction procedure of the *sppA* deficient strain was described briefly as following. First, the upstream (a) and downstream (b) regions of *sppA* were amplified respectively by the corresponding primers sppA-KF1/sppA-KR1 and sppA-KF2/sppA-KR2 listed in Additional file 1: Table S1, and fused by splicing overlap extension (SOE)-PCR using the primers sppA-KF1/sppA-KR2. The fused fragments were cloned into T_2_(2)-Ori at the restriction sites *Xba*I/*Sac*I, diagnostic PCR and DNA sequencing confirmed that the vector, named T_2_-sppA, was constructed successfully (Fig. 1).

Then, the vector T_2_-sppA was electro-transferred into *B. licheniformis* BL10 and was verified by diagnostic PCR and plasmids extractions [22, 23]. The positive transformants were cultivated in the LB liquid medium with 20 μg/mL kanamycin at 45 °C, and subcultured for three generations, then transferred into kanamycin-free LB medium at 37 °C for another six generations. The cells were then plated on LB agar medium with or without kanamycin and incubated for 20 h, respectively. The primers sppA-KYF/sppA-KYR were used for PCR to verify the double crossover strains, and DNA sequencing confirmed that the *sppA* deficient strain (*B. licheniformis* BL10S) was constructed successfully. The *tepA* deficient strain (*B. licheniformis* BL10T) was constructed using methods similar to those employed for the construction of *B. licheniformis* BL10S.

### Construction of the protein expression vector

The nattokinase expression vector used in this study was obtained from our previously reported research [7], and the α-amylase expression vector was constructed according to the following method. P43 promoter was from *B. subtilis* 168. The gene *amyL* (FJ556804.1) coding for α-amylase (containing its own signal peptide and terminator) was amplified from the genome DNA of *B. licheniformis* WX-02 with the corresponding primers (Additional file 1: Table S1), purified and fused by SOE-PCR, and inserted into the expression vector pHY300PLK at the restriction sites *Eco*RI and *Xba*I, plasmid extraction and DNA sequencing have confirmed that this new plasmid was constructed successfully, named pHY-amyL. The green fluorescent protein (GFP) expression vector (pHY-GFP) was constructed by using a similar method.

### Gene integration in *B. licheniformis*

To develop strains to over express *sppA* and *tepA,* the individual genes *sppA* and *tepA* mediated by P43 promoter were integrated into the genome of *B. licheniformis* by the previous reported method [8]. The resulting strains *B. licheniformis* BL10GS, capable of overexpressing *sppA* and *B. licheniformis* BL10GT, capable of overexpressing *tepA*, were verified by PCR and used for further assays.

#### Enzyme activity assay

Two proteins, nattokinase and α-amylase, were served as the target proteins to evaluate the efficiency of host strains constructed in this research, and the methods for activity assay were described previously [8].

#### α-amylase activity assay

The mixture of 20 mL soluble starch solution (2 g/mL) and 5 mL 0.12 M phosphate buffer (pH 6) was added into the 50 mL centrifuge tube, and incubated at 60 °C for 8 min. The fermentation supernatant at a volume of 1 mL was added into the tube and incubated at 60 °C for another 10 min. Then, the 1-mL reaction solution was transferred into a new centrifuge tube containing 0.5 mL 0.1 mol/L HCl and 5 mL dilute iodine solution (266 mM KI and 0.35 mM I_2_), and the absorbance was detected under 660 nm wavelength. The mixture containing 0.5 mL 0.1 mol/L HCl and 5 mL dilute iodine solution was served as the control. The α-amylase activity was calculated by the standard curve made from the different concentrations of starch solution (0.00, 0.10, 0.20, 0.30, 0.40, 0.50, 0.60, 0.70, 0.80, 0.90, 1.00 mg/mL). One Unit of α-amylase activity (U) was defined as the amount of enzyme for catalyzing 1 mg starch to release reducing sugar (glucose) per minute at 60 °C (pH 6) [24].

#### Nattokinase activity assay

The nattokinase activity was measured according to our previous reported method [8]: the mixture of 0.4 mL fibrinogen solution (0.72%, w/v) and 1.4 mL Tris–HCl (50 mM, pH 7.8) was incubated in a test tube at 37 °C for 10 min, followed by adding 0.1 mL thrombin solution (20 U/mL) to form the fibrin at 37 °C for 10 min. Then, the diluted sample solution (D) at the volume of 0.1 mL was added into the tube and shaken at 37 °C for 60 min at the interval of every 15 min, followed by adding 2 mL trichloroacetic acid (TCA) solution (0.2 M) to stop the reaction (AT). As for the control group, the mixture of 0.1 mL sample solution and 2 mL TCA solution (0.2 M) was used after incubating at 37 °C for 60 min (AB). Finally, all mixtures were centrifuged at 12000*g* for 10 min, and the absorbance of the supernatant was determined at 275 nm. One unit of nattokinase activity (1 FU) was defined as the amount of enzyme leading to the 0.01 increase for A_275_ in 1 min, and the formula was as follows:

$$ {\text{Nattokinase activity }}\left( {{\text{FU}}/{\text{mL}}} \right) = \left( {{\text{AT}} - {\text{AB}}} \right) \times 100 \times {\text{D}}/ 6 $$

### Determination of the total extracellular protein and target protein concentrations

To determine the extracellular protein concentration, the volume of 100 μL fermentation broth was mixed with an equal volume of Coomassie Brilliant Blue G-250 and the absorbance was measured at 595 nm. The concentration of total extracellular protein was calculated by the standard curve made from different concentrations of bovine serum albumin (BSA) [25]. Meanwhile, the cells were washed three times with physiological saline, followed with sonication (pulse: 1 s on; 2 s off; total 4 min) to disrupt cells, and the concentration of intracellular protein was determined with the similar method.

For determining the target protein concentration, the mixture of 900 μL fermentation broth with 100 μL TCA (6.12 M) was maintained at 4 °C overnight, and centrifuged at 13,000*g* for 10 min. The precipitate was washed three times with ethanol and dried, and re-dissolved in a solution containing 2 M thiourea and 8 M urea. The sample was then mixed with equal volume of 2× SDS-PAGE loading buffer, and the volume of 10 μL solution was subjected to SDS-PAGE analysis. BSA was used as the standard. Protein concentration of the sample was calculated by comparing the area and pixel counts of the bands imaged from the gel with that of the standard, using a Bio-Rad GS-800 calibrated densitometer and Quantity One software [26].

### Analysis of transcription level

The total RNA of the strain was extracted by the TRIzol^®^ Reagent (Invitrogen, USA) according to our previously reported method [20], and contaminant DNA was digested by the Rnase-free DNase I enzyme (TaKaRa, Japan). The RevertAid First Strand cDNA Synthesis Kit (Thermo, USA) was applied to amplify the first stand of cDNA. The primers in the (seeing the Additional file: Table S2) were used for amplifying the corresponding genes in the 10 μL Real-Time PCR mixture (containing 5 μL SYBR^®^ Select Master Mix, 0.5 μL primers, 1 μL cDNA, 3.5 μL DEPC water), and 16S rRNA from *B. licheniformis* BL10 was used as the reference gene to normalize the data. The Vii7 Real-Time PCR system (ABI, USA) was used for the Real-Time PCR reaction (95 °C for 3 min; 40 cycles of 95 °C for 30 s, 60 °C for 30 s). The transcriptional levels for genes in the recombinant strain were compared with those of the control strain after normalization to the reference gene 16S rRNA, and the experiments were performed in triplicate.

### Statistical analyses

All samples were analyzed in triplicate, and the data were presented as the mean ± the standard deviation for each sample point. All data were conducted to analyze the variance at P < 0.05 and P < 0.01, and a *t* test was applied to compare the mean values using the software package Statistica 6.0 [27].

## Results

### Establishment of the signal peptide peptidase gene deficient strains

Based on the genome sequence and annotation of *B. licheniformis* WX-02, two genes (*sppA* and *tepA*) were predicted to function as SPPs in *B. licheniformis* WX-02 [28]. In order to investigate the function of these gene products, *sppA* and *tepA* were deleted in the parent strain *B. licheniformis* BL10, respectively. Figure 1 shows the construction procedure of the *sppA* knockout vector, and the bands amplified from mutants had the same length of the homologous arms without that of target genes (Additional file 1: Figure S1), confirming that *sppA* was deleted successfully. The new strain was named *B. licheniformis* BL10S. Similarly, *tepA* was also deleted using a similar method, and the resultant strain was named *B. licheniformis* BL10T.

### Effects of signal peptide peptidase deficiency on the total extracellular protein secretion

**Fig. 2:**
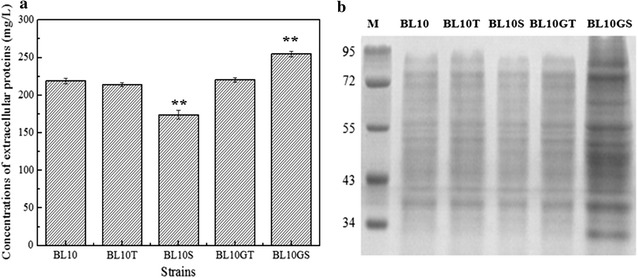
Effects of deficiency or over-expression of *sppA* and *tepA* on the extracellular secretion. **a** The concentrations of total extracellular proteins of different strains; **b** SDS-PAGE analysis of the extracellular proteins from different host strains. *M* protein marker (95, 72, 55, 43, 34 kDa); *Lane 1 B. licheniformis* BL10; *Lane 2 B. licheniformis* BL10T; *Lane 3 B. licheniformis* BL10S; *Lane 4 B. licheniformis* BL10GT; *Lane 5 B. licheniformis* BL10GS. Data are represented as the means of three replicates and *bars* represent the standard deviations, *P < 0.05; and **P < 0.01 indicate the significance levels between recombinant strains and control strain

The *sppA* and *tepA* deficient strains, as well as the parental strain BL10, were cultivated in ME media for 24 h, respectively, and the concentrations of total extracellular proteins were assayed to evaluate the importance of these genes on total extracellular protein secretion. As shown in Fig. 2a, the yield of total extracellular proteins decreased by 35% in the *sppA* deficient strain, compared to BL10. Meanwhile, there was no difference for the concentrations of total extracellular proteins produced by the *tepA* deficient strain and BL10, and these results were positively correlated with the SDS-PAGE results in Fig. 2b, which suggested that *sppA* gene product had the strongest effect on protein secretion in *B. licheniformis*.

### Effects of signal peptide peptidase deficiency on the target protein production

In this study, nattokinase and α-amylase were selected as the target proteins to evaluate the function of *sppA* and *tepA*. Nattokinase expression vector pP43SacCNK was made in a previous study [7], and the α-amylase expression vector pHY-amyL harboring P43 promoter (K02174.1), *amyL* (FJ556804.1) coding for α-amylase (containing its own signal peptide and terminator) was verified by DNA sequence. The pP43SacCNK and pHY-amyL plasmids were individually electro-transferred into the parental strain *B. licheniformis* BL10, as well as the SPP gene deficient strains BL10S and BL10T. PCR verification and plasmid extraction confirmed that the recombinant strains were constructed successfully.

**Fig. 3:**
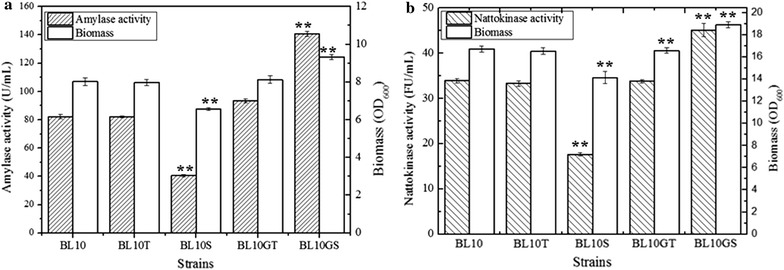
The activities of target proteins and biomass of different host strains harboring pHY-amyL or pP43SacCNK. **a** α-Amylase activities and biomass of different host strains harboring pHY-amyL; **b** nattokinase activities and biomass of different host strains harboring pP43SacCNK. Data are represented as the means of three replicates and* bars* represent the standard deviations, *P < 0.05; and **P < 0.01 indicate the significance levels between recombinant strains and control strain

**Fig. 4:**
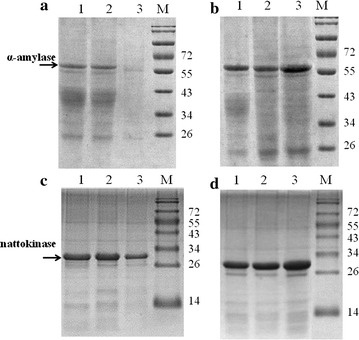
SDS-PAGE analysis of the extracellular proteins from different host strains harboring pHY-amyL or pP43SacCNK. **a** SDS-PAGE analysis of extracellular proteins from *tepA* and *sppA* deficient strains harboring pHY-amyL, *Lane 1* BL10/pHY-amyL; *Lane 2* BL10T/pHY-amyL; *Lane 3* BL10S/pHY-amyL. **b** SDS-PAGE analysis of extracellular proteins from *tepA* and *sppA* overexpression strains harboring pHY-amyL, *Lane 1* BL10/pHY-amyL; *Lane 2* BL10GT/pHY-amyL; *Lane 3* BL10GS/pHY-amyL; **c** SDS-PAGE analysis of extracellular proteins from the *tepA* and *sppA* deficient strains harboring pP43SacCNK, *Lane 1* BL10/pP43SacCNK; *Lane 2* BL10T/pP43SacCNK; *Lane 3* BL10S/pP43SacCNK; **d** SDS-PAGE analysis of extracellular proteins from *tepA* and *sppA* overexpression strains harboring pP43SacCNK, *Lane 1* BL10/pP43SacCNK; *Lane 2* BL10GT/pP43SacCNK; *Lane 3* BL10GS/pP43SacCNK. *M* Protein marker (120, 100, 95, 72, 55, 43, 34, 26, 14 kDa)

Furthermore, the extracellular activities of α-amylase and nattokinase produced by different strains were measured. As shown in Fig. 3, the extracellular activities of α-amylase and nattokinase were decreased by 51 and 48%, and the maximum biomass was decreased by 18 and 15% respectively due to the deficiency of *sppA*. Furthermore, the specific activities of α-amylase and nattokinase in BL10S were 17011.86 U/g_DCW_ and 3436.71 FU/g_DCW_ (1 OD_600_ = 0.363 g_DCW_/L), which were 40 and 38% lower compared with those of BL10 (28238.63 U/g_DCW_ and 5585.39 FU/g_DCW_), respectively. There were no differences in the yields of the target proteins and specific enzyme activities between the *tepA* deficient strain BL10T and BL10. These results suggest that SppA and not TepA, plays an important role for protein secretion. Additionally, the deficiency of *sppA* might not only affect the target protein yields in *B. licheniformis*, but also the cell biomass was decreased in the *sppA* deficient strains (Fig. 4a, c).

### Effects of overexpression of signal peptide peptidase on the protein secretion

To verify the vital role of SPP in extracellular protein production, the genes *sppA* and *tepA* mediated by P43 promoter were further overexpressed in *B. licheniformis* BL10 by inserting the relevant expression cassettes into the chromosome by homologous recombination, respectively [8]. The *sppA* insert was verified by PCR and the bands amplified from the mutant strain exhibited the same length exactly as the length combination of the homologous arms and the *sppA* gene plus P43 promoter. Furthermore, DNA sequencing results confirmed the successful integration of *sppA* into *B. licheniformis* BL10, resulting in the new strain *B. licheniformis* BL10GS. The *tepA* overexpression strain was constructed and verified using the similar methods. The resulting new strain was named *B. licheniformis* BL10GT.

Both SPP overexpression strains BL10GS and BL10GT were cultivated in the ME medium for 24 h. As shown in Fig. 2a, the yield of total extracellular proteins increased by 37% in the *sppA* overexpression strain BL10GS, compared with that of the parental strain BL10. These results positively correlated with the SDS-PAGE results in Fig. 2b. Meanwhile, overexpression of *tepA* had no effect on the concentration of the total extracellular proteins. Furthermore, in order to analyze the influence of *sppA* overexpression on cell lysis, the green fluorescent protein (GFP) was expressed in the parental strain BL10, the *sppA* mutant strain BL10S and the *sppA* overexpression strain BL10GS (Additional file 1: Figure S2). As shown in Additional file 1: Figure S3, GFP was expressed successfully in these strains, and no GFP was detected in ME medium after cultivation for 24 h. These results suggest that overexpression of *sppA* has no effect on the cell lysis. Furthermore, these results indicate that SppA could be used to improve the concentration of extracellular proteins produced by *B. licheniformis*.

The nattokinase and α-amylase expression vectors were electro-transferred into the *sppA* and *tepA* overexpression strains, and the recombinant strains were cultivated in the nattokinase and amylase production media for 48 h to evaluate the effects of overexpression of SPP genes on the target protein production, respectively. As shown in Fig. 3, the activities of target enzymes and cell yields both increased in the *sppA* overexpression strain, which was consistent with the improvement of target protein concentrations (Table 2). Based on our results, the cell biomass of *sppA* deficiency strains were decreased by 16 and 13% during α-amylase and nattokinase production, and the decreases in cell biomass observed were much lower than decreases in protein yields (67 and 30%, respectively). Also, the specific activities of α-amylase and nattokinase were increased by 43 and 18% respectively in the *sppA* overexpression strain, compared with those produced by *B. licheniformis* BL10. These results indicate that overexpression of *sppA* improves both cell biomass and protein yields. Meanwhile, in the *tepA* overexpression strain, only a 12% improvement of specific α-amylase activity was obtained and overexpression of *tepA* had no effect on the concentrations of nattokinase (Fig. 4b, d). These results were consistent with the enzymes activities (Fig. 3). Moreover, the concentrations of intracellular protein were measured in these strains duringα-amylase and nattokinase production, the SPPs deficiency and overexpression have no effects on the concentrations of intracellular protein (Additional file 1: Table S3).

**Table 2:** Effects of deletion or overexpression of *sppA* and *tepA* on the concentrations of target proteins

	Strains	BL10	BL10T	BL10S	BL10GT	BL10GS
Concentrations (mg/L)	pHY-amyL	89.04 (± 5.43)	86.93 (± 6.25)	46.78 (± 2.43)	104.29 (± 9.54)	147.95 (± 11.32)
	pP43SacCNK	215.30 (± 10.19)	205.41 (± 21.64)	108.94 (± 8.11)	212.62 (± 14.46)	281.19 (± 12.65)

### Effect of SppA on the α-amylase secretion and cell growth during the fermentation process

**Fig. 5:**
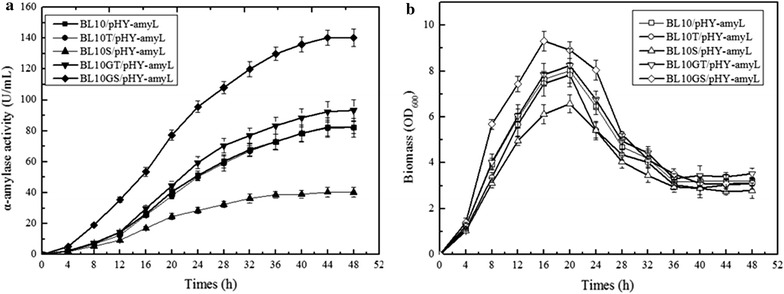
Fermentation process curves of α-amylase production by different host strains harboring pHY-amyL. **a** α-Amylase activities; **b** OD_600_

Figure 5 shows the time courses of the cell growth and α-amylase yield of *B. licheniformis* BL10/pHY-amyL, *B. licheniformis* BL10S/pHY-amyL, *B. licheniformis* BL10T/pHY-amyL, *B. licheniformis* BL10GT/pHY-amyL, and *B. licheniformis* BL10GS/pHY-amyL in the α-amylase production medium. The culture was sampled every 4 h, and α-amylase activity and biomass were measured at the defined time. Based on our results, the α-amylase activities of BL10GS/pHY-amyL were higher than those of BL10/pHY-amyL and BL10S/pHY-amyL throughout the whole fermentation process, and the α-amylase activity was the highest in *B. licheniformis* BL10GS/pHY-amyL and the lowest in BL10S/pHY-amyL. Also, the biomass of BL10GS/pHY-amyL was higher than those of other strains throughout the fermentation process. Meanwhile, deletion or overexpression of *tepA* had no effect on the cell growth and target production during the production of α-amylase (Fig. 5). These results indicated that overexpression of *sppA* could not only improve the protein secretion level, but also enhance the biomass yield.

**Fig. 6:**
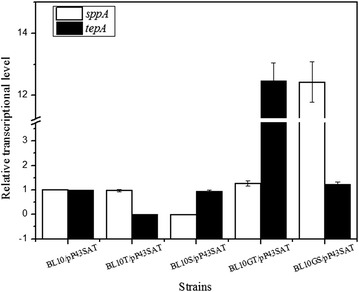
Transcriptional levels of *sppA* and *tepA* in the different strains during α-amylase production

The relative gene expression levels of BL10/pHY-amyL, BL10T/pHY-amyL, BL10S/pHY-amyL, BL10GT/pHY-amyL and BL10GS/pHY-amyL were also evaluated at the mid-log phase during the α-amylase production process (8 h). As shown in Fig. 6, the transcriptional expression levels of *sppA* and *tepA* could not be detected in the *sppA* and *tepA* deficient strains, and the *sppA* and *tepA* transcriptional expression levels increased by 11.43- and 11.47-fold in the BL10GS and BL10GT strains, respectively compared to the parental strain.

## Discussion

Heterologous expression is an effective strategy to improve the target protein production, and many strategies have been carried out to improve the protein yield [3, 9, 29–31]. It has been previously shown that SPP can play an important role during protein secretion [32], However, prior to the current study, no research has been reported on the improvement of protein secretion by overexpression of SPP. In this study, we have demonstrated that the SppA SPP plays an important role for protein secretion in *B. licheniformis*, and overexpression of *sppA* could improve the yields of the total extracellular proteins and target proteins.

In this study, the yields and specific activities of target proteins were decreased significantly in the *sppA* deficient strain, and they were increased in the *sppA* overexpression strain, compared with those of the parental strain BL10. However, different expression levels of an alternative SPP, TepA, had no effect on the concentrations of heterologously produced extracellular target proteins. These results suggest that SppA is very important for protein secretion in *B. licheniformis*, and are consistent with previously reported results in *B. subtilis* [18, 19]. Previously, several researches have implied that the TepA of *B. subtilis* is a cytoplasmic, ClpP-like germination protease involved in spore outgrowth, and it is expressed almost exclusively during sporulation, and researches proposed that the TepA of *B. subtilis* is not the SPP [33, 34]. Since *B. licheniformis* have high homology with *B. subtilis*, it was suggested that the TepA of *B. licheniformis* BL10 might also not be the SPP by our results. Meanwhile, SPPs are required for degradation of signal peptides that are inhibitory to protein translocation [35, 36], and the deficiency of *sppA* might influence the ability to remove remnant signal peptides in the cellular membrane. It has been proposed that the accumulation of these remnant signal peptides might block the membrane channel for protein translocation and hinder protein secretion [18]. Therefore, we postulated that overexpression of *sppA* would increase the removal of the remnant signal peptides, therefore improving protein secretion. The extracellular activities of α-amylase produced by *B. licheniformis* BL10GS/pHY-amyL were higher than those of BL10/pHY-amyL throughout the whole fermentation process. These results indicate that overexpression of *sppA* could improve the α-amylase yield from the beginning of the fermentation, and SppA is necessary for highly efficient protein secretion. Meanwhile, the concentrations of intracellular protein showed no differences among these recombinant strains, which indicated that the higher levels of extracellular proteins might be due to the increase level of recombinant proteins production. Thus, in-depth research on remnant signal peptides in the cell membrane should be carried out to further understand this mechanism. In addition, our results indicate that overexpression of *sppA* could improve the cell growth (Fig. 3), which partially contributed to the increase of the α-amylase activity. However, since the increase in cell biomass observed was much lower than the increase in the α-amylase activity, we concluded that the enhanced α-amylase activity was mainly due to the increased protein secretion.

In order to improve extracellular protein production, several strategies have been developed for signal peptide processing by manipulating the signal peptidase and signal peptidase [37]. Malten et al. [29] have overexpressed the type I signal peptidase SipM in *Bacillus megaterium* MS941, and the yield of target protein Dsrs, mediated by its own signal peptide, was increased by 3.7-fold. Also, the signal peptidase I SipV was confirmed to play a vital role for nattokinase (mediated by the signal peptide of AprE) production in our previous study [8]. Previous studies have shown that either signal peptidase I (SipS or SipT) was sufficient for protein secretion [38]. Signal peptidases serve as the “scissors” to cut off the signal peptides from the pre-proteins [39], and multiple signal peptidases were responsible for the cleavage of different signal peptides [40]. Therefore, overexpression of a single signal peptidase could not be used as a universal strategy for enhancing protein secretion. In this research, the yield of total extracellular proteins was improved markedly in the *sppA* overexpression strain, and the concentrations of nattokinase and α-amylase mediated by different signal peptides were increased by 30 and 67%, respectively. These results implied that overexpression of *sppA* might act as an efficient and useful strategy for protein production.

In conclusion, this study implied that SppA is the main functional SPP for protein secretion in *B. licheniformis*, and overexpression of *sppA* indeed improved the concentrations of target proteins, biomass and specific activities. In this study, overexpression of *sppA* might act as a useful and efficient strategy to improve protein secretion. The host strain *B. licheniformis* BL10GS demonstrated high efficiency for protein secretion and could be employed as a promising industrial strain for extracellular production of valuable proteins.

## Additional file

**Additional file 1.** All the sequences of the primers and concentrations of total intracellular proteins were listed in the Additional file (Table S1, Table S2 and Table S3). The agarose gel electrophoresis analysis of the SPPs deficient strains and the fluorescence detection of the cell and the fermentation supernatant of the BL10/pHY-GFP, BL10S/pHY-GFP, BL10GS/pHY-GFP were also contained in the Additional file (Fig. S1, Fig. S2 and Fig. S3). This information is available free of charge via the Internet http://microbialcellfactories.biomedcentral.com/.

## References

[1] Yang H, Liu L, Shin HD, Chen RR, Li J, Du G, Chen J (2013). Comparative analysis of heterologous expression, biochemical characterization optimal production of an alkaline alpha-amylase from alkaliphilic *Alkalimonas amylolytica* in *Escherichia coli* and *Pichia pastoris*. Biotechnol Prog.

[2] Lin S, Zhang M, Liu J, Jones GS (2015). Construction and application of recombinant strain for the production of an alkaline protease from *Bacillus licheniformis*. J Biosci Bioeng.

[3] Wang P, Wang P, Tian J, Yu X, Chang M, Chu X, Wu N (2016). A new strategy to express the extracellular alpha-amylase from *Pyrococcus furiosus* in *Bacillus amyloliquefaciens*. Sci Rep.

[4] Westers L, Westers H, Quax WJ (2004). *Bacillus subtilis* as cell factory for pharmaceutical proteins: a biotechnological approach to optimize the host organism. Biochim Biophys Acta.

[5] van Dijl JM, Hecker M (2013). *Bacillus subtilis*: from soil bacterium to super-secreting cell factory. Microb Cell Fact.

[6] Pohl S, Harwood CR (2010). Heterologous protein secretion by *Bacillus* species from the cradle to the grave. Adv Appl Microbiol.

[7] Wei X, Zhou Y, Chen J, Cai D, Wang D, Qi G, Chen S (2015). Efficient expression of nattokinase in *Bacillus licheniformis*: host strain construction and signal peptide optimization. J Ind Microbiol Biotechnol.

[8] Cai D, Wei X, Qiu Y, Chen Y, Chen J, Wen Z, Chen S (2016). High-level expression of nattokinase in *Bacillus licheniformis* by manipulating signal peptide and signal peptidase. J Appl Microbiol.

[9] Kang Z, Yang S, Du G, Chen J (2014). Molecular engineering of secretory machinery components for high-level secretion of proteins in *Bacillus* species. J Ind Microbiol Biotechnol.

[10] Wang Y, Liu Y, Wang Z, Lu F (2014). Influence of promoter and signal peptide on the expression of pullulanase in *Bacillus subtilis*. Biotechnol Lett.

[11] Bolhuis A, Broekhuizen CP, Sorokin A, van Roosmalen ML, Venema G, Bron S, Quax WJ, van Dijl JM (1998). SecDF of *Bacillus subtilis*, a molecular Siamese twin required for the efficient secretion of proteins. J Biol Chem.

[12] Hunt JF, Weinkauf S, Henry L, Fak JJ, McNicholas P, Oliver DB, Deisenhofer J (2002). Nucleotide control of interdomain interactions in the conformational reaction cycle of SecA. Science.

[13] Chen J, Fu G, Gai Y, Zheng P, Zhang D, Wen J (2015). Combinatorial Sec pathway analysis for improved heterologous protein secretion in *Bacillus subtilis*: identification of bottlenecks by systematic gene overexpression. Microb Cell Fact.

[14] Chen J, Gai Y, Fu G, Zhou W, Zhang D, Wen J (2015). Enhanced extracellular production of alpha-amylase in *Bacillus subtilis* by optimization of regulatory elements and over-expression of PrsA lipoprotein. Biotechnol Lett.

[15] Fu LL, Xu ZR, Li FW, Shuai JB, Lu P, Hu CX (2007). Protein secretion pathways in *Bacillus subtilis*: implication for optimization of heterologous protein secretion. Biotechnol Adv.

[16] Harwood CR, Cranenburgh R (2008). *Bacillus* protein secretion: an unfolding story. Trends Microbiol.

[17] van Roosmalen ML, Geukens N, Jongbloed JD, Tjalsma H, Dubois JY, Bron S, van Dijl JM, Anne J (2004). Type I signal peptidases of Gram-positive bacteria. Biochim Biophys Acta.

[18] Bolhuis A, Matzen A, Hyyrylainen HL, Kontinen VP, Meima R, Chapuis J, Venema G, Bron S, Freudl R, MaartenvanDijl J (1999). Signal peptide peptidase- and ClpP-like proteins of *Bacillus subtilis* required for efficient translocation and processing of secretory proteins. J Bio Chem..

[19] Nam SE, Paetzel M (2013). Structure of signal peptide peptidase A with C-termini bound in the active sites: insights into specificity, self-processing, and regulation. Biochemistry.

[20] Qiu Y, Xiao F, Wei X, Wen Z, Chen S (2014). Improvement of lichenysin production in *Bacillus licheniformis* by replacement of native promoter of lichenysin biosynthesis operon and medium optimization. Appl Microbiol Biotechnol.

[21] Qi G, Kang Y, Li L, Xiao A, Zhang S, Wen Z, Xu D, Chen S (2014). Deletion of meso-2,3-butanediol dehydrogenase gene *budC* for enhanced D-2,3-butanediol production in *Bacillus licheniformis*. Biotechnol Biofuels.

[22] Xue GP, Johnson BP, Dalrymple BP (1999). High osmolarity improves the electro-transformation efficiency of the gram-positive bacteria *Bacillus subtilis* and *Bacillus licheniformis*. J Microbiol Meth.

[23] Qiu Y, Zhang J, Li L, Wen Z, Nomura CT, Wu S, Chen S (2016). Engineering *Bacillus licheniformis* for the production of meso-2,3-butanediol. Biotechnol Biofuels.

[24] Chen J, Zhou Y, Zhao X, Chen S, Wei X (2015). Comparative analysis of different *Bacillus licheniformis* host strains on the secretion expression of α-amylase. Food Sci.

[25] Grintzalis K, Georgiou CD, Schneider YJ (2015). An accurate and sensitive Coomassie Brilliant Blue G-250-based assay for protein determination. Anal Biochem.

[26] Voigt B, Schweder T, Sibbald MJ, Albrecht D, Ehrenreich A, Bernhardt J, Feesche J, Maurer KH, Gottschalk G, van Dijl JM, Hecker M (2006). The extracellular proteome of *Bacillus licheniformis* grown in different media and under different nutrient starvation conditions. Proteomics.

[27] Tian G, Fu J, Wei X, Ji Z, Qi G, Chen S (2013). Enhanced expression of *pgdS*, gene for high production of poly-γ-glutamic aicd with lower molecular weight in *Bacillus licheniformis* WX-02. J Chem Technol Biotechnol.

[28] Yangtse W, Zhou Y, Lei Y, Qiu Y, Wei X, Ji Z, Qi G, Yong Y, Chen L, Chen S (2012). Genome sequence of *Bacillus licheniformis* WX-02. J Bacteriol.

[29] Malten M, Nahrstedt H, Meinhardt F, Jahn D (2005). Coexpression of the type I signal peptidase gene *sipM* increases recombinant protein production and export in *Bacillus megaterium* MS941. Biotechnol Bioeng.

[30] Degering C, Eggert T, Puls M, Bongaerts J, Evers S, Maurer KH, Jaeger KE (2010). Optimization of protease secretion in *Bacillus subtilis* and *Bacillus licheniformis* by screening of homologous and heterologous signal peptides. Appl Environ Microbiol.

[31] Zhang JK, Kang Z, Ling Z, Cao W, Liu L, Wang M, Du G, Chen J (2013). High-level extracellular production of alkaline polygalacturonate lyase in Bacillus subtilis with optimized regulatory elements. Bioresour Technol.

[32] Nam SE, Kim AC, Paetzel M (2012). Crystal structure of *Bacillus subtilis* signal peptide peptidase A. J Mol Biol.

[33] Westers H, Darmon E, Zanen G, Veening JW, Kuipers OP, Bron S, Quax WJ, Van Dijl JM (2004). The *Bacillus* secretion stress response is an indicator for alpha-amylase production levels. Lett Appl Microbiol.

[34] Traag BA, Pugliese A, Setlow B, Setlow P, Losick R (2013). A conserved ClpP-like protease involved in spore outgrowth in *Bacillus subtilis*. Mol Microbiol.

[35] Chen CY, Malchus NS, Hehn B, Stelzer W, Avci D, Langosch D, Lemberg MK (2014). Signal peptide peptidase functions in ERAD to cleave the unfolded protein response regulator XBP1u. EMBO J.

[36] Voss M, Schroder B, Fluhrer R (2013). Mechanism, specificity, and physiology of signal peptide peptidase (SPP) and SPP-like proteases. Biochim Biophys Acta.

[37] Dalbey RE, Wang P, van Dijl JM (2012). Membrane proteases in the bacterial protein secretion and quality control pathway. Microbiol Mol Biol Rev.

[38] Tjalsma H, Bolhuis A, van Roosmalen ML, Wiegert T, Schumann W, Broekhuizen CP, Quax WJ, Venema G, Bron S, van Dijl JM (1998). Functional analysis of the secretory precursor processing machinery of *Bacillus subtilis*: identification of a eubacterial homolog of archaeal and eukaryotic signal peptidases. Genes Dev.

[39] Auclair SM, Bhanu MK, Kendall DA (2012). Signal peptidase I: cleaving the way to mature proteins. Protein Sci.

[40] Song Y, Nikoloff JM, Zhang D (2015). Improving protein production on the level of regulation of both expression and secretion pathways in *Bacillus subtilis*. J Microbiol Biotechnol.

