# Supplementary material for: A novel strategy to improve protein secretion via overexpression of the SppA signal peptide peptidase in Bacillus licheniformis
Source: Microb Cell Fact. 2017 Apr 24;16:70. doi: 10.1186/s12934-017-0688-7 (PMC5404308; doi:10.1186/s12934-017-0688-7)
Supplement: Supplementary file 1 — Additional file 1. All the sequences of the primers and concentrations of total intracellular proteins were listed in the Additional file (Table S1, Table S2 and Table S3). The agarose gel electrophoresis analysis of the SPPs deficient strains and the fluorescence detection of the cell and the fermentation supernatant of the BL10/pHY-GFP, BL10S/pHY-GFP, BL10GS/pHY-GFP were also contained in the Additional file (Fig. S1, Fig. S2 and Fig. S3). This information is available free of charge via the Internet http://microbialcellfactories.biomedcentral.com/. [file 12934_2017_688_MOESM1_ESM.docx]

A Novel Strategy to Improve Protein Secretion via Overexpression of the SppA Signal Peptide Peptidase in *Bacillus licheniformis*

Dongbo Cai ^a#^, Hao Wang ^a#^, Penghui He ^a^, Chengjun Zhu ^a^, Qin Wang ^a^, Xuetuan Wei ^b^, Christopher T. Nomura ^a, c^, Shouwen Chen ^a*^

^a^ *Hubei Collaborative Innovation Center for Green Transformation of Bio-Resources，College of Life Sciences, Hubei University, Wuhan 430062, PR China*

*^b^ College of Food Science and Technology*, *Huazhong Agricultural University, Wuhan 430070, China*

*^c^ Department of Chemistry, The State University of New York College of Environmental Science and Forestry (SUNY ESF), Syracuse, NY 13210 USA*

^#^ These authors contributed equally to this article

*Corresponding author: Prof. Shouwen Chen

Tel./fax.: +86 027-88666081.

*E-mail address*: [hzauskycai@126.com(D](mailto:hzauskycai@126.com(D). Cai); [18071729546@163.com](mailto:18071729546@163.com) (H. Wang); [penghuihehubu@163.com](mailto:penghuihehubu@163.com) (P. He); [zhuchengjun@gmail.com](mailto:zhuchengjun@gmail.com) (C. Zhu); [qinwang327@gmail.com](mailto:qinwang327@gmail.com) (Q. Wang); [weixuetuan@mail.hzau.edu.cn](mailto:weixuetuan@mail.hzau.edu.cn) (X. Wei); ctnomura@esf.edu (CT. Nomura); [mel212@126.com](mailto:mel212@126.com) (S. Chen).

*Postal address*: No. 368 Youyi Avenue, Wuchang District, Wuhan 430062, Hubei, PR China

| Primer name | Sequence 5′→3′ |
| --- | --- |
| sppA-KF1 | GGCGAGCTCAGGCAGTCATCCCTGAAGTTCGTCT |
| sppA-KR1 | CCTTTTTCATCGCTTTGACCGCAGTTTCTTCCTGGGTGTCATTG |
| sppA-KF2 | CAATGACACCCAGGAAGAAACTGCGGTCAAAGCGATGAAAA |
| sppA-KR2 | GCTCTAGAGACAAGCGACACGACCTTCAGCCCG |
| sppA-KYF | GAACGGATGGATACTTCATTTAGAC |
| sppA-KYR | GGATAACCGCAGTGTCCGCAAAGAA |
| tepA-KF1 | GGCGAGCTCCCTGATTGAAAAAGGAGATGTCG |
| tepA-KR1 | CGAGTTTTCCGCCTGCCCTGCGAAGGCTGCTCCCCTTGGCTGT |
| tepA-KF2 | GACAGCCAAGGGGAGCAGCCTTCGCAGGGCAGGCGGAAAAC |
| tepA-KR2 | TGCTCTAGATTCTCCTCGTCAAAAAACTGGGTGT |
| tepA-KYF | GCCCAGGTCGTATTCGCACAAAAAC |
| tepA-KYR | AATAAAAGGGCTCCAATCATTCCGC |
| GsppA-F | GACTAGTAGAAATAAATGTTTCGGCGGT |
| GsppA-R | CGAGATCTGTCAAGCAGAAAAGCAAACAGC |
| GtepA-F | GACTAGTCGCCTTAAAACAGACGATGCGTG |
| GtepA-R | CGAGATCTGCATATTTATCCTTGAGACACTCC |
| P43-F | GGAATTCTGATAGGTGGTATGTTTTCG |
| P43-R | CCACAATTTTTTGCTTCTCACTTCATGTGTACATTCCTCTC |
| TamyL-F | GTACAAGCAGCTGCACAATAAAAGAGCAGAGAGGACGGATT |
| TamyL-R | GCTCTAGAGCCGCAATAATGCCGTCGCACTG |
| pHY-F | GTTTATTATCCATACCCTTAC |
| pHY-R | CAGATTTCGTGATGCTTGTC |
| T2-F | ATGTGATAACTCGGCGTA |
| T2-R | GCAAGCAGCAGATTACGC |

**Table S1 The primers used in this research**

**Table S2 The primers used for RT-qPCR in this research**

| Primer name | Sequence 5′→3′ |
| --- | --- |
| sppA-F | TCTGCCGTAAAGGGCATTGT |
| sppA-R | GCTTTCCATGATGACCCCGA |
| tepA-F | TCCAGCTTCCTCCCCAAAAC |
| tepA-R | AGCATTTCCGCAATCGCAAG |
| 16S-F | TCAGCTCGTGTCGTGAGAT |
| 16S-R | CGATCCGAACTGAGAACAG |

**Table S3** Effects of deletion or overexpression of *sppA* and *tepA* on the concentrations of total intracellular proteins.

|  | Strains | BL10 | BL10T | BL10S | BL10GT | BL10GS |
| --- | --- | --- | --- | --- | --- | --- |
| Concentrations  (mg/L) | pHY-amyL | 103.43 (±5.43) | 105.32 (±6.25) | 104.28 (±5.32) | 104.79 (±6.27) | 102.95 (±7.14) |
|  | pP43SacCNK | 115.32 (±6.04) | 112.43 (±9.54) | 113.87 (±4.09) | 114.25 (±7.53) | 111.54 (±7.28) |

**Fig. S1 Double exchanged strain was screened by PCR. A: Double exchanged of *sppA* deficient strain.** Lane1: PCR product of the genome DNA of BL10S by primers sppA-KYF/sppA-KYR (1591 bp); Lane2: PCR product of the genome of BL10 by primers sppA-KYF/sppA-KYR as control (2088 bp); M: DL5000 Marker (5000 bp, 3000 bp, 2000 bp, 1500 bp, 1000 bp, 750 bp, 500 bp, 250 bp); **B:** **Double exchanged of *tepA* deficient strain.** Lane1: PCR product of the genome DNA of BL10T by primers tepA-KYF/tepA-KYR (1511 bp); Lane2: PCR product of the genome of BL10 by primers tepA-KYF/tepA-KYR as control (2046 bp); M: DL5000 Marker (5000 bp, 3000 bp, 2000 bp, 1500 bp, 1000 bp, 750 bp, 500 bp, 250 bp)


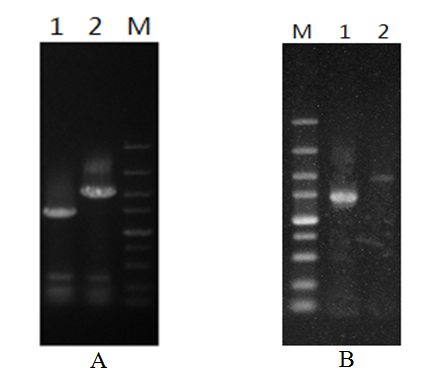


**Fig. S1**

**Fig. S2 The fermentation curves of BL10/pHY-GFP, BL10S/pHY-GFP, BL10GS/pHY-GFP in the ME medium.**


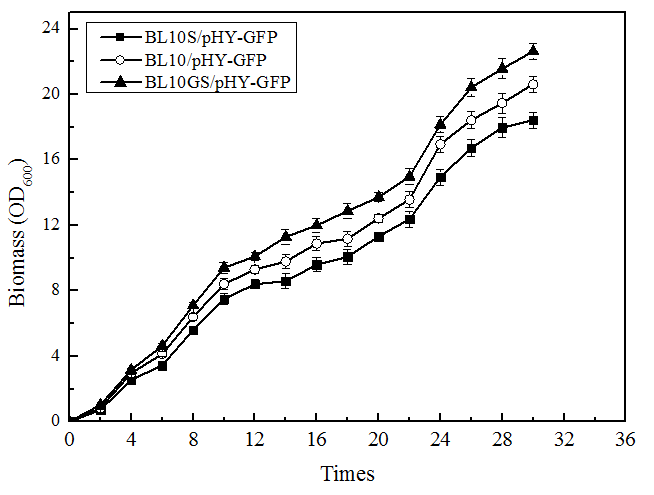


**Fig. S2**

**Fig. S3 Fluorescence detection of the cell and fermentation supernatant of BL10/pHY-GFP, BL10S/pHY-GFP, BL10GS/pHY-GFP**

**
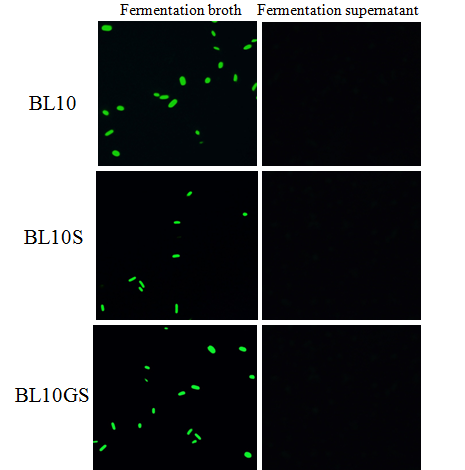
**

**Fig. S3**
